# Multi-dimensional mismatch and barriers for promoting PrEP among men who have sex with men in China: a cross sectional survey from the Demand-side

**DOI:** 10.1186/s12981-022-00497-6

**Published:** 2023-02-13

**Authors:** Yuan Guan, Tangkai Qi, Qibin Liao, Renfang Zhang, Jun Chen, Li Liu, Yinzhong Shen, Han Zhu, Qi Tang, Hongzhou Lu

**Affiliations:** 1 School of Public Health, Fudan University, Shanghai, China; 2 National Clinical Research Center for Infectious Disease Shenzhen Third Peoples Hospital The Second Affiliated Hospital School of Medicine, Southern University of Science and Technology, Shenzhen, Guangdong China; 3 Department of Infection and Immunity, Shanghai Public Health Clinical Center, Fudan University, Shanghai, China

**Keywords:** PrEP, MSM, HIV, Risk behaviors, HIV prevention

## Abstract

**Background:**

Men who have sex with men (MSM) is a key population for preventing HIV in China, yet pre-exposure prophylaxis (PrEP) is not widely accepted in this population. The objective of this manuscript was to assessed the barriers in the acknowledgement and uptake focusing the demand side.

**Methods:**

An online questionnaire survey was conducted from December 2018 to January 2019. All participants were required to scan two-dimensional code which was the online crowdsourcing survey platform to complete the electronic questionnaire anonymously.

**Results:**

Among 1915 MSM from thirty-four cities of China, 512 (26.7%) versus 1617 (84.4%) had an objective or subjective need of PrEP, respectively. One hundred and six (5.5%) reported affordability and only 23 (1.2%) had ever taken it. Age, living alone and occupation were associated with the objective needs. Age, income, sexual behavior were associated with actual usage. The participants who they had objective need to use PrEP are the population which we should focus on.

**Conclusion:**

A wide disconnect exists among the objective need, willingness, affordability and uptake of PrEP. Cost was the most prevalent barrier, accounting for 78.22% of individuals who needed and wished for PrEP but finally failed to receive it. The findings might facilitate optimizing future allocation of resources to better promote PrEP in Chinese MSM.

**Supplementary Information:**

The online version contains supplementary material available at 10.1186/s12981-022-00497-6.

## Introduction

Men who have sex with men (MSM) is a key population for preventing the HIV epidemic [1, 2]. In China, the HIV prevalence of MSM has increased from 1.5% in 2005 to 8.0% in 2015, compared to 0.037% in the general population [3–11]. Since 2010, Chinese MSM have remained the highest infection rates among all key populations which contributed to over a quarter of newly reported HIV/AIDS cases [12]. In light of these trends, there is an urgent need for China to implement HIV prevention measures among MSM.

HIV prevention strategies mainly include behavioral strategies (condom use, abstinence seroadaptive strategies), and chemoprophylactic strategies (pre-exposure prophylaxis, post-exposure prophylaxis). And consistent condom use remains the primary HIV prevention approach targeted to MSM; however, only 48.8% of Chinese MSM report using condoms consistently [13]. Pre-exposure prophylaxis (PrEP) is regarded as a promising HIV prevention strategy by using antiretroviral drugs (ARVs), which could reduce the possibility of HIV infection [14, 15]. Three types of PrEP are available, including daily oral PrEP, on-demand PrEP (taking ARV before and after sex) and long-acting injectable PrEP (injecting long-acting drugs every 4–12 weeks). The iPrEx (Pre-exposure Prophylaxis Initiative) study proclaimed that the HIV infection rate was reduced by 44% overall in this groundbreaking clinical trial [16]. While the medication is clearly effective, the fact remains that its success is ultimately dependent on its adoption.

The World Health Organization (WHO) has issued guidelines recommending oral PrEP as part of HIV combination prevention approaches to prevent HIV among key populations since 2014 [17]. The latest Chinese ARV guidelines and expert consensus statement also call for delivering quality PrEP services to MSM [18]. Along with PrEP promotion programs, awareness and willingness of PrEP uptake have increased in some regions over the past several years, but still modest in most countries [19]. Poverty and drugs constraints are identified barriers to accessing to HIV prevention and treatment services; the cost is a major barrier to PrEP implementation even in Western Europe high-income countries [20]. Tenofovir/emtricitabine (TDF/FTC) is available in China at a price of some $310/month, if the patients take PrEP daily. In addition, cultural beliefs about health, HIV stigma, homophobia and limited health insurance coverage for PrEP are further barriers to PrEP uptake [21]. Some studies have reported the acknowledgement and actual uptake of PrEP are still low in Chinese MSM [22, 23]. There is an urgent need to investigate the key barriers for promoting PrEP in China.

In this large internet-based online survey, we multi-dimensionally analyzed the current characteristics and influence factors of PrEP among MSM in China. Then assessed the common barriers in the acknowledgement and uptake focusing the demand side, as well as discussing their possible underlying factors.

## Materials and methods

### Study designing

This research was a cross-sectional study designed to investigate the objective need, willingness, affordability, actual uptake of PrEP as HIV prevention and associated factors among Chinese MSM. The study was organized by Shanghai Public Health Clinical Centre (SPHCC), a designated medical institution that provides outpatient and inpatient care for HIV/AIDS patients in Shanghai as well as East China, which is the important sites for initial screening and linkage to PrEP services. The Centre has limited capacity to contact with a large number of HIV-negative MSM, and therefore relies on a non-governmental organization (NGO) to conduct the questionnaire survey. This NGO working for MSM used a design involving snowball sampling since MSM in China are a marginalized and stigmatized group which can be difficult to reach. Via WeChat (a Chinese cell/web application for messaging, social media, communications—including answering questionnaires), the NGO successfully sent a unique two-dimensional code linking the electronic questionnaire to potential participants. All participants signed an electronic informed consent form and then filled in an electronic questionnaire, designed on the basis of the Canadian guideline on HIV pre-exposure prophylaxis and nonoccupational post-exposure prophylaxis (2018) [24]. Experienced physicians and nurses were involved in the development of the questionnaire to ensure that participants could adequately understand the information. This study was approved by the institutional review board of SPHCC (No. 2018-S043-01). Informed consent was obtained from all participants.

### Participants and setting

Between December 2018 and January 2019, an internet-based survey was administrated on PrEP knowledge and acceptability among Chinese MSM. The participants were required to scan two-dimensional code which was link to the online crowdsourcing survey platform to complete the electronic questionnaire anonymously. For eligibility, participants have to be at least 18 years of age, be able to sign consents, and being MSM. HIV-positive patients, dyslexic people and foreigners were excluded from our study population. All were informed that participation was voluntary and anonymous by clicking a button to read the online consent form. To avoid repetition, questionnaires bearing the same IP address as previously submitted questionnaires were not accepted.

### Procedures

The questionnaires were developed to obtain the information about demographics, sexual behaviors, perceived HIV risk, the assessment of objective need of PrEP, willingness and obstacles to use PrEP, affordability and usage of PrEP among Chinese MSM.

Our primary objective was to estimate the proportion of participants who are ‘‘optimal’’ candidates for initial PrEP scale-up, defined as “objective need of PrEP”. To be specific, the objective HIV risk was defined as MSM who reported condomless anal sex within the last 6 months and who had any of the following [24]: (*i*) Infectious syphilis or rectal bacterial sexually transmitted infection (STI), particularly if diagnosed in the preceding 12 months; (*ii*) Recurrent use of nonoccupational post-exposure prophylaxis (nPEP) (more than once); (*iii*) Ongoing sexual relationship with HIV-positive partner with substantial risk of transmissible HIV; or (*iv*) High-incidence risk index (HIRI)-MSM risk score  ≥ 11 (Additional file 1: Table S1).

Willingness to use PrEP was defined as answering ‘‘agree’’ or ‘‘strongly agree’’ on a five-point Likert-type scale in response to the statement, ‘‘I would be interested in taking PrEP to reduce my current risk of HIV infection, if cost is not a factor.’’ To gain further insight into willingness to use, our questionnaires included an open-ended question asking ‘‘What concerns do you have about PrEP?’’ Responses were coded and reported for those not willing to use PrEP.

The on-demand PrEP was estimated to cost an average of $150/month, since the average consumption of was around half daily demand of TDF/FTC as to the literature [25]. Affordability of PrEP was defined as who could have PrEP insurance coverage or pay the full cost of medications ($150/month) out-of-pocket. The cut-off value was set at $150/month; fees are set mainly on the basis of two ARVs.

Uptake of PrEP was designed to ask “How many times have you had a prescription for PrEP from your doctor in the last 6 months?”.

The target sample size was calculated based on the number of respondents needed to estimate the primary proportion of interest with reasonable precision using the equation n = Z^2^1-α/2 *p(1-p)/d^2^, which Z^2^1-α/2 is the 1-α/2 critical value of the standard normal distribution; p is the proportion of interest and d is half the length of the desired 95% confidence interval. Using a preliminary estimate of p = 0.7 based on our prior study in which 70% of MSM described ‘‘definitely’’ or ‘‘maybe’’ being willing to use PrEP. And after allowing for 10% incomplete responses, we estimated roughly 400 responses would be required. This study ultimately returned 1,915 questionnaires, which was an adequate sample size.

### Data analysis

All statistical analyses were performed using SPSS (Version 19.0). Differences were assessed by *t* test, ANOVA, chi-square test; Fisher's exact test was used for variables as appropriate. Univariate and multivariate logistic regression analyses were performed separately to examine the factors associated with objective need of PrEP, willingness to use PrEP, affordability of PrEP and uptake of PrEP. Univariate regression analysis was performed at first, followed by multiple logistic regression analysis including those with a *p* < 0.05 in univariate analysis. Odd ratios (ORs) and 95% confidence intervals (95% CI) were calculated.

## Results

### Sample current characteristics

MSM from 34 cities of China participated in the survey, and total 1915 participants enrolled in the final analysis. As shown in the Table 1, the majority were aged 21–30 years old (77.1%), with 17.0% aged 20 years or younger. Approximately one-in-five of participants lived in the central region of China, and half of in Eastern China. And a total of 621 participants (32.4%) reported they were living alone. High levels of education and employment were evident among the sample, which more than three-quarters of participants (80.7%) had attended college and only 87 people (4.5%) were unemployed. A total of 1253 participants (65.4%) had annual income of less than $7,500. A small sample (8.3%) had over 6 male anal sex partners in the past 6 months and (2.3%) had male sex partners with HIV positive. Additionally, almost a third of participants reported they had condomless anal sex within the last 6 months.

**Table 1:** Participant characteristics of 1915 cases enrolled in this study

Items	Total (*n* = 1915)	Objective need of PrEP events (*n* = 512)	Willingness to use PrEP events (*n* = 1617)	Affordability of PrEP events (*n* = 106)	Uptake of PrEP events (*n* = 23)
Age (years), mean (SD)	23.8 (4.4)	27.0 (2.1)	23.7 (4.1)	23.8 (5.0)	24.0 (3.5)
Age group [*n* (%)]					
≤ 20	325 (17.0%)	60 (11.7%)	270 (16.7%)	26 (24.5%)	4 (17.4%)
21–30	1476 (77.1%)	423 (82.6%)	1254 (77.6%)	70 (66.0%)	19 (82.6%)
31–40	98 (5.1%)	25 (4.9%)	79 (4.9%)	8 (7.5%)	0 (0%)
≥ 45	16 (0.8%)	4 (0.8%)	14 (0.9%)	2 (1.9%)	0 (0%)
Residential region [*n* (%)]					
Eastern China	1121 (58.5%)	304 (59.4%)	956 (59.1%)	55 (51.9%)	15 (65.2%)
Central China	391 (20.4%)	111 (21.7%)	320 (19.8%)	28 (26.4%)	4 (17.4%)
Western China	403 (21.0%)	97 (18.9%)	341 (21.1%)	23 (21.7%)	4 (17.4%)
Solitary [*n* (%)]	621 (32.4%)	147 (28.7%)	524 (32.4%)	35 (33.0%)	7 (30.4%)
Education [*n* (%)]					
Middle school or below	13 (0.7%)	4 (0.8%)	7 (0.4%)	0 (0%)	0 (0%)
High school or equal	357 (18.6%)	108 (21.1%)	300 (18.6%)	15 (14.2%)	4 (17.4%)
Undergraduate	1297 (67.7%)	331 (64.6%)	1108 (68.5%)	78 (73.6%)	14 (60.9%)
Graduate or above	248 (13.0%)	69 (13.5%)	202 (12.5%)	13 (12.3%)	5 (21.7%)
Occupation [*n* (%)]					
Students	1041 (54.4%)	234 (45.7%)	887 (54.9%)	57 (53.8%)	12 (52.2%)
Employees	787 (41.1%)	256 (50.0%)	663 (41.0%)	42 (39.6%)	11 (47.8%)
Unemployed	87 (4.5%)	22 (4.3%)	67 (4.1%)	7 (6.6%)	0 (0%)
Annual income ($) [*n* (%)]					
< 7500	1253 (65.4%)	318 (62.1%)	1056 (65.3%)	66 (62.3%)	10 (43.5%)
7500–15,000	407 (21.3%)	121 (23.6%)	339 (21.0%)	14 (13.2%)	6 (26.1%)
15,000–30,000	181 (9.5%)	55 (10.7%)	158 (9.8%)	18 (17.0%)	2 (8.7%)
30,000–450,000	44 (2.3%)	11 (2.1%)	38 (2.4%)	2 (1.9%)	4 (17.4%)
> 450,000	30 (1.6%)	7 (1.4%)	26 (1.6%)	6 (5.7%)	1 (4.3%)
No. male anal sex partners in the past 6 months [*n* (%)]					
0–5	1756 (91.7%)	426 (83.2%)	1472 (91.0%)	100 (94.3%)	12 (52.2%)
6–10	107 (5.6%)	54 (10.5%)	95 (5.9%)	4 (3.8%)	7 (30.4%)
> 10	52 (2.7%)	32 (6.3%)	50 (3.1%)	2 (1.9%)	4 (17.4%)
Had male sex partners with HIV positive [*n* (%)]	44 (2.3%)	12 (2.3%)	39 (2.4%)	3 (2.8%)	1 (4.3%)
HIRI-MSM risk score, mean (SD)	11.52 (5.59)	19.4 (4.1)	11.7 (5.7)	11.6 (6.2)	17.1 (7.6)
Having condomless anal sex within the last 6 months [*n* (%)]	577 (30.1%)	512 (100%)	505 (31.2%)	34 (32.1%)	12 (52.2%)

*SD* standard deviation, *No.* number of, *HIRI-MSM* High-incidence risk index

Of the total 1915 participants, 512 (26.7%) had objective need to use PrEP; 1617 (84.4%) were willing to use PrEP; 106 (5.5%) participants reported that they could afford PrEP and only 23 (1.2%) had actual uptake of PrEP for HIV prevention.

**Fig. 1:**
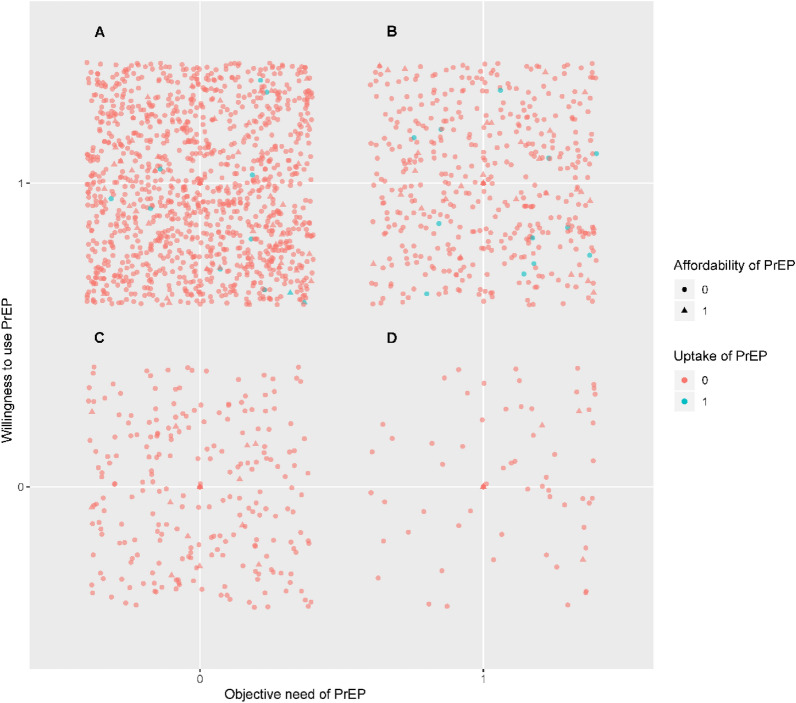
The current characteristics of PrEP among MSM in China. Of the total 1915 participants, 512 (26.7%) had objective need to use PrEP, 1617 (84.4%) were willing to use PrEP, 106 (5.5%) participants reported that they could afford PrEP and only 23 (1.2%) had actual uptake of PrEP for HIV prevention. Area A represents those who are willing to use PrEP but do not have objective need, while Area B represents those who are willing to use PrEP and objectively need it. It is clear that most of the MSM population has a high willingness to use PrEP, but may not actually be the top candidates for PrEP promotion. It is also noteworthy that only 5.5% of the MSM in our study could afford PrEP, and even fewer actually used it

From Fig. 1, we could notice that Area A represents those who are willing to use PrEP but without objective need, while Area B represents those who have willing and objective need. It is clear that most of the MSM have a high willingness to use PrEP, but may not actually be the top candidates for PrEP promotion. It is also noteworthy that only 5.5% of the MSM in our study could afford cost of PrEP, and fewer used it actually. This suggests that there is a large gap between the demand for PrEP services and the actual use of PrEP among MSM.

### Factors associated with current characteristics of PrEP multi-dimensionally among MSM in China

#### Influence factors associated with objective need of PrEP for HIV prevention

Overall, 512 (26.7%) participants were investigated that they had objective need to use PrEP for HIV prevention. There were higher proportions of objective need to use PrEP among those who were aged range from 21 to 30 (82.6%), lived in eastern China (59.4%), had undergraduate education or above (78.1%), had an annual income of less than $7,500 (62.1%) and had 0–5 male anal sex partners in the past 6 months (83.2%).

Univariate analysis indicated that significant variables associated with objective need to use PrEP included age, solitary, occupation. In multivariate logistic regression model, participants who were aged range from 21 to 30 (OR = 1.47; 95% CI 1.06–2.03), employees (OR = 1.73; 95% CI 1.37–2.19) were significantly more likely to need PrEP for HIV prevention, whereas those reporting living alone were less likely to need PrEP (OR = 0.67; 95% CI 0.54–0.85) (Table 2).

**Table 2:** Influence factors associated with Objective need of PrEP for HIV prevention

Items	Univariate *P-*value	OR (95%CI)	Multivariate *P*-value	OR (95%CI)
Age (years)	0.14	1.02 (0.99–1.04)	–	–
Age group				
≤ 20	0.003	1	0.034	1
21–30	< 0.0001	1.77 (1.31–2.40)	0.019	1.47 (1.06–2.03)
31–40	0.13	1.51 (0.89–2.58)	0.89	0.96 (0.54–1.71)
≥ 45	0.52	1.47 (0.46–4.72)	0.96	0.97 (0.30–3.20)
Residential region			–	–
Eastern China	0.35	1		
Central China	0.63	1.07 (0.83–1.38)		
Western China	0.23	0.85 (0.66–1.11)		
Solitary	0.036	0.79 (0.63–0.99)	0.001	0.67 (0.54–0.85)
Education			–	–
Middle school or below	0.32	1		
High school or equal	0.97	0.98 (0.29–3.24)		
Undergraduate	0.67	0.77 (0.24–2.52)		
Graduate or above	0.82	0.87 (0.26–2.91)		
Occupation				
Students	< 0.0001	1	< 0.0001	1
Employees	< 0.0001	1.66 (1.35–2.05)	< 0.0001	1.73 (1.37–2.19)
Unemployed	0.55	1.17 (0.71–1.93)	0.62	1.14 (0.68–1.90)
Annual income ($)			–	–
< 7500	0.34	1		
7500–15,000	0.08	1.24 (0.97–1.59)		
15,000–30,000	0.15	1.28 (0.91–1.81)		
30,000–450,000	0.96	0.98 (0.49–1.96)		
> 450,000	0.80	0.90 (0.38–2.11)		

*SD* standard deviation, *OR* Odd ratio

#### Influence factors associated with Willingness to use PrEP for HIV prevention

Not surprisingly, 1617 (84.4%) participants reported that they were willing to use PrEP for HIV prevention.

Univariate analysis indicated that significant variables included education, occupation, number of male anal sex partners in the past 6 months, HIRI-MSM risk score and condomless anal sex within the last 6 months. In multivariate analysis, those who had high school or above education were significantly more willing to use PrEP for HIV prevention, whereas those were unemployed were significantly less willing to use PrEP (OR = 0.54; 95% CI 0.32–0.93) (Table 3).

**Table 3:** Influence factors associated with Willingness to use PrEP for HIV prevention

Items	Univariate *P-*value	OR (95%CI)	Multivariate *P*-value	OR (95%CI)
Age (years)	0.08	0.98 (0.95–1.00)	–	–
Age group			–	–
≤ 20	0.58	1		
21–30	0.40	1.15 (0.83–1.59)		
31–40	0.57	0.85 (0.48–1.51)		
≥ 45	0.65	1.43 (0.32–6.45)		
Residential region			–	–
Eastern China	0.27	1		
Central China	0.11	0.78 (0.57–1.06)		
Western China	0.75	0.95 (0.69–1.30)		
Solitary	0.96	0.99 (0.76–1.29)	–	–
Education				
Middle school or below	0.016	1	0.023	1
High school or equal	0.009	4.51 (1.46–13.92)	0.012	4.47 (1.40–14.28)
Undergraduate	0.004	5.03 (1.67–15.12)	0.007	4.80 (1.53–15.04)
Graduate or above	0.022	3.76 (1.21–11.73)	0.036	3.52 (1.09–11.35)
Occupation				
Students	0.13	1	0.08	1
Employees	0.57	0.93 (0.72–1.20)	0.46	0.91 (0.70–1.18)
Unemployed	0.04	0.58 (0.34–0.99)	0.027	0.54 (0.32–0.93)
Annual Income ($)			–	–
< 7500	0.78	1		
7500–15,000	0.64	0.93 (0.69–1.26)		
15,000–30,000	0.29	1.28 (0.81–2.04)		
30,000–450,000	0.71	1.18 (0.49–2.83)		
> 450,000	0.72	1.21 (0.42–3.51)		
No. male anal sex partners in the past 6 months				
0–5	0.04	1	0.15	1
6–10	0.18	1.53 (0.83–2.82)	0.53	1.24 (0.64–2.39)
> 10	0.03	4.82 (1.17–19.94)	0.06	4.13 (0.94–18.06)
Had male sex partners with HIV positive	0.44	1.45 (0.57–3.71)	–	–
HIRI-MSM risk score	< 0.0001	1.05 (1.02–1.07)	0.13	1.03 (0.99–1.08)
Having condomless anal sex within the last 6 months	0.015	1.43 (1.07–1.90)	0.82	1.06 (0.67–1.65)

*No.* number of, *HIRI-MSM* High-incidence risk index, *OR* Odd ratio

#### Influence factors associated with affordability of PrEP for HIV prevention

Only 106 (5.5%) participants were investigated to indicate that they could afford PrEP for HIV prevention.

Univariate logistic regression model indicated that significant variables included age and annual income. In multivariate analysis, those who had annual income between 15,000–30,000$ (OR = 2.22; 95% CI 1.24–4.00) or over 45,000$ (OR = 4.56; 95% CI 1.68–12.38) were significantly likely to afford PrEP, whereas those were aged range from 21–30 were significantly less possible to afford PrEP (OR = 0.54; 95% CI 0.33–0.89) (Table 4).

**Table 4:** Influence factors associated with Affordability of PrEP for HIV prevention

Items	Univariate *P-*value	OR (95%CI)	Multivariate *P*-value	OR (95%CI)
Age (years)	0.99	1.00 (0.96–1.05)	–	–
Age group				
≤ 20	0.045	1	0.10	1
21–30	0.019	0.57 (0.36–0.91)	0.015	0.54 (0.33–0.89)
31–40	0.96	1.02 (0.45–2.34)	0.48	0.71 (0.28–1.80)
≥ 45	0.53	1.64 (0.35–7.62)	0.76	0.78 (0.15–4.06)
Residential region			–	–
Eastern China	0.24	1		
Central China	0.94	1.50 (0.93–2.39)		
Western China	0.53	1.17 (0.71–1.94)		
Solitary	0.89	1.03 (0.68–1.56)	–	–
Education			–	–
Middle school or below	–	–		
High school or equal	0.55	0.79 (0.37–1.70)		
Undergraduate	0.64	1.16 (0.63–2.12)		
Graduate or above	0.62	1		
Occupation			–	–
Students	0.58	1		
Employees	0.90	0.97 (0.65–1.47)		
Unemployed	0.32	1.51 (0.67–3.42)		
Annual income ($)				
< 7500	0.001	1	0.001	1
7500–15,000	0.14	0.64 (0.36–1.15)	0.32	0.74 (0.40–1.36)
15,000–30,000	0.014	1.99 (1.15–3.43)	0.008	2.22 (1.24–4.00)
30,000–450,000	0.83	0.86 (0.20–3.62)	0.93	0.94 (0.22–4.06)
> 450,000	0.002	4.50 (1.78–11.38)	0.003	4.56 (1.68–12.38)
No. male anal sex partners in the past 6 months			–	–
0–5	0.60	1		
6–10	0.40	0.64 (0.23–1.78)		
> 10	0.57	0.66 (0.16–2.76)		
Had male sex partners with HIV positive	0.71	1.26 (0.38–4.12)	–	–
HIRI-MSM risk score	0.81	1.00 (0.97–1.04)	–	–
Having condomless anal sex within the last 6 months	0.65	1.10 (0.72–1.68)	–	–

*No.* number of, *HIRI-MSM* High-incidence risk index *OR* Odd ratio

#### Influence factors associated with uptake of PrEP for HIV prevention

Finally, only 23 (1.2%) participants reported that they had actual uptake of PrEP for HIV prevention in the past 6 months.

Univariate analysis indicated that significant variables included annual income, number of male anal sex partners in the past 6 months, HIRI-MSM risk score and condomless anal sex within the last 6 months. In multivariate analysis, those who had annual income between 30,000–45,000$ (OR = 10.55; 95% CI 2.88–38.66) or had 6–10 male anal sex partners in the past 6 months (OR = 4.90; 95% CI 1.56–15.40) were more likely to had actual uptake of PrEP (Table 5).

**Table 5:** Influence factors associated with Uptake of PrEP for HIV prevention

Items	Univariate *P-*value	OR (95%CI)	Multivariate *P*-value	OR (95%CI)
Age (years)	0.79	1.01 (0.93–1.10)	–	–
Age group			–	–
≤ 20	1.00	1		
21–30	0.94	1.05 (0.35–3.10)		
31–40	–	–		
≥ 45	–	–		
Residential region			–	–
Eastern China	0.81	1		
Central China	0.63	0.76 (0.25–2.31)		
Western China	0.59	0.74 (0.24–2.24)		
Solitary	0.84	0.91 (0.37–2.23)	–	–
Education			–	–
Middle school or below	–	–		
High school or equal	0.38	0.55 (0.15–2.07)		
Undergraduate	0.23	0.53 (0.19–1.49)		
Graduate or above	0.68	1		
Occupation			–	–
Students	0.90	1		
Employees	0.64	1.22 (0.53–2.77)		
Unemployed	–	–		
Annual Income ($)				
< 7500	0.001	1	0.008	1
7500–15,000	0.23	1.86 (0.67–5.15)	0.23	1.88 (0.67–5.32)
15,000–30,000	0.67	1.39 (0.30–6.39)	0.81	1.21 (0.54–5.78)
30,000–450,000	< 0.0001	12.43 (3.74–41.33)	< 0.0001	10.55 (2.88–38.66)
> 450,000	0.17	4.29 (0.53–34.60)	0.21	3.94 (0.46–33.99)
No. male anal sex partners in the past 6 months				
0–5	< 0.0001	1	0.024	1
6–10	< 0.0001	10.17 (3.92–26.40)	0.007	4.90 (1.56–15.40)
> 10	< 0.0001	12.11 (3.77–38.92)	0.12	3.53 (0.72–17.39)
Had male sex partners with HIV positive	0.52	1.96 (0.26–14.83)	–	–
HIRI-MSM risk score	< 0.0001	1.14 (1.08–1.20)	0.08	1.11 (0.99–1.25)
Having condomless anal sex within the last 6 months	0.025	2.56 (1.12–5.84)	0.60	0.67 (0.15–3.00)

*No.* number of, *HIRI-MSM* High-incidence risk index *OR* Odd ratio

### The barriers of MSM with objective need of PrEP for HIV prevention

**Fig. 2:**
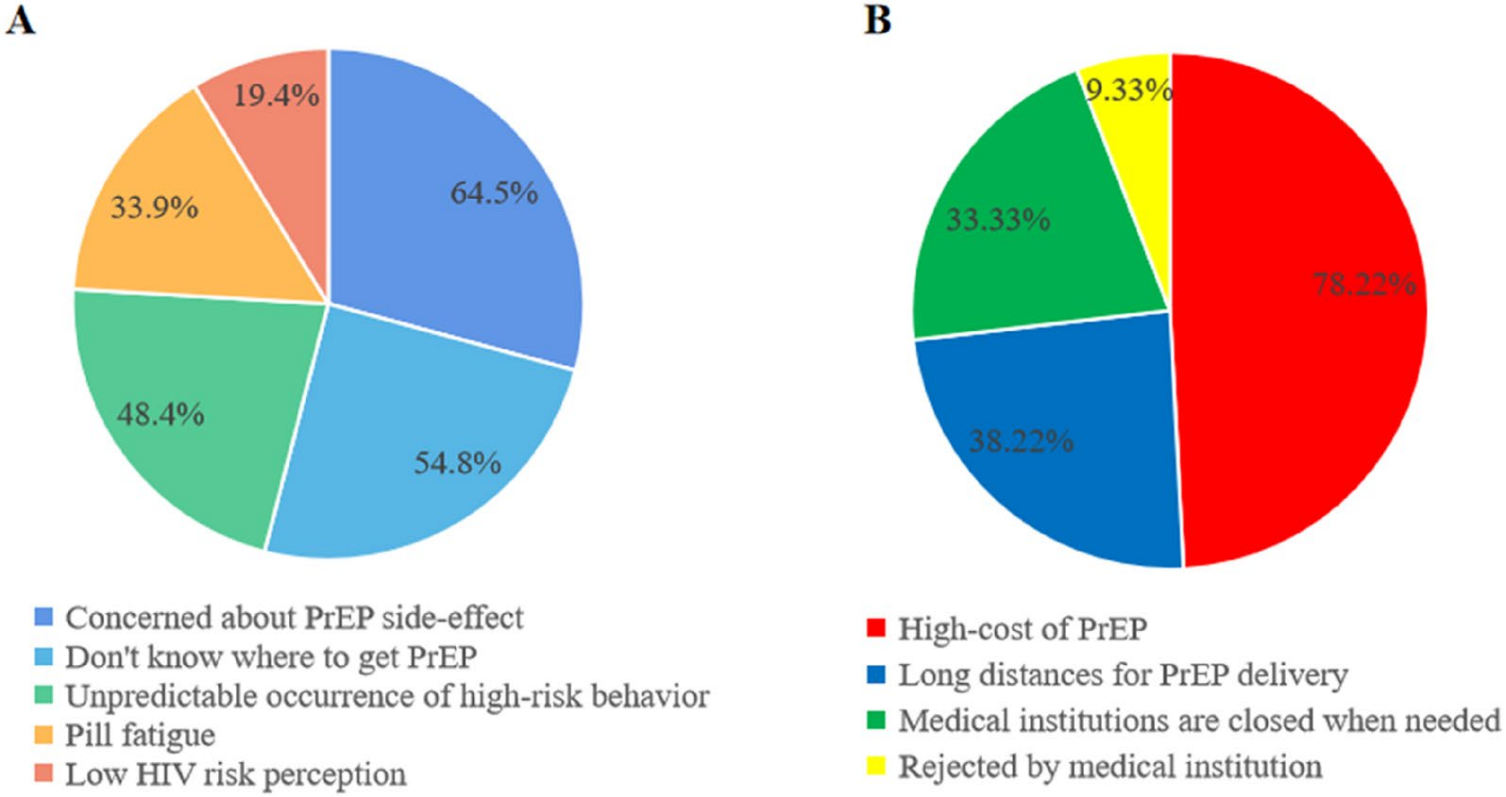
Subjective and objective obstacles of MSM with objective need of PrEP for HIV prevention. Of the 62 participants who had objective need but unwillingness to use PrEP, the reasons were summarized as below: 40 (64.5%) were concerned about PrEP side-effect; 34 (54.8%) did not know where to get PrEP; 30 (48.4%) believed the occurrence of high-risk behavior is unpredictable; 12 (19.35%) had low HIV risk perception and 21 (33.87%) had pill fatigue. Of the 450 participants who failed to obtain PrEP but with willingness, the reasons were summarized as below: 352 (78.2%) considered the cost of PrEP is too high; 172 (38.2%) worried about long distances for PrEP delivery; 150 (33.33%) reported that medical institutions were closed when needed, and 42 (9.33%) were previously rejected by medical institution

The participants who had objective need to use PrEP for HIV prevention are the population that this study focus on. Therefore, we investigated the subjective and objective barriers of them to use PrEP for HIV prevention. Of the only 62 participants who had objective need but unwillingness to use PrEP, the main reasons were summarized as worrying about ARVs side-effect and lack of access to PrEP. It is noteworthy that the majority (87.9%) participants who failed to obtain PrEP but with willingness, the main reason was summarized as concerning about cost of PrEP (Fig. 2).

## Discussion

This study contributes to the literature on PrEP acceptability and affordability by examining in the willingness and actual uptake of PrEP among Chinese MSM. Among the 1915 participants from across the country, covering a range of ages, education, occupation and income levels; 512 (26.7%) were defined as having objective need for their HIV prevention using PrEP according to the guideline [24]. This indicates substantial need of PrEP among MSM in China, given its huge population base. However, only 1.2% of the participants reported actual uptake of PrEP for HIV prevention. The data was similar to previous researches where actual uptake of PrEP in Chinese MSM is low [26–32]. Our analysis indicated non-solitary, on job and aged from 21 to 30 years were associated with higher possibility of objective demand of PrEP. Those who are young, employed or living in a group should be better covered in future PrEP programs in Chinese MSM. In this way, social-economical and medical resource might be distributed more effectively to benefit those who are in need.

Participants in this study showed high willingness to use PrEP. The reality was that only a small proportion of MSM who were willing to use PrEP for HIV prevention actually used it. There is a significant gap between the willingness and actual uptake of PrEP among MSM, which may be attributed to subjective or objective barriers. Subjective barriers included low education level or unemployment. This calls for more educating and promoting programs for PrEP in less educated or unemployed MSM in China. The major objective barriers included high-cost of PrEP, long distances for drug delivery and medical institutions closing when needed. With the release of Chinese ARV guidelines and expert consensus on PrEP, there is an increasing number of hospitals providing service on PrEP [34]. The mismatch between service time and expected time calls for more service available at the patients convenience, such as weekend time. Notably, not all MSM who willing to use PrEP have objective need to use it. In the future, PrEP promoting strategies should focus on those with actual need.

Still, affordability held a key barrier against a final decision to initiate PrEP in our study, which was coherent with previous reports [26, 35, 36]. The participants reported high initial willingness to use PrEP (when it was free) but low pay willingness, and this discrepancy might be consequence of PrEP cost and affordability. More than three quarters of participants (84.4%) selected that they should have taken PrEP, if cost were not a factor. Compared to low-income MSM, our study identified those who had higher annual income had higher possibility of PrEP affordability and uptake. Overall, only 5.5% of participants were willing to pay 150$ monthly for PrEP, which equals to the price of Truvada used on demand. It may reflect a more realistic level of PrEP acceptability among Chinese MSM. Those earns less than $15,000 annually could hardly afford the cost of PrEP. A recent article revealed affordable PrEP enabled a sustainable low HIV incidence of 1 to 2 per 1000 person-years among MSM in Australia [37]. This highlighted the importance of affordable and available service of PrEP for controlling the HIV epidemic. Potential strategies might include but not limited to price negotiation with the drugmakers, fincaial subsidy, insurance coverage, generic agents, reward for compliance and on-demand PrEP.

Our study was drawn from a large and targeted data set covering most geographical areas in China. However, there were also several limitations, and the most important of which were the use of a cross-sectional survey and its natural reliance on self-reported information. The data was collected online platform and likely biased towards young MSM, potentially limiting the generalizability of our findings. Face-to-face interview will be needed in future studies to better cover the aged population. Second, the cross-sectional design without a specific sampling frame led to a convenience sample, so that no causal relationship could be established. The snowball sampling could not ensure equal representation, so not all MSM had similar access to HIV services across these 34 sites. Finally, self-report bias might exist as we asked the participants to recall their STI history and sexual behaviors within 6 months. To reduce such bias, we emphasized to the participants that participation was totally voluntary, and participants were guaranteed that their personal data was highly confidential and inaccessible to third parties.

Despite these limitations, this national survey illustrated a critical mismatch between high demand and low uptake of PrEP among MSM in China. It also provided direction to address the key challenges in promoting PrEP, including screening priority populations, developing affordable drug strategies. Further work is needed to optimize strategies for determining PrEP eligibility, bridging the disconnect between objective and subjective HIV risk, and improving engagement in the PrEP cascade.

## Conclusion

In conclusion, there is a wide disconnect among “objective need of PrEP”, “willingness to use PrEP”, “affordability of PrEP” and “uptake of PrEP” in Chinese MSM. It revealed key barriers in the acknowledgement and practice of PrEP in this population, such as cost, education, age, occupation, behaviors and habitation patterns. Findings on the low willingness to pay for PrEP indicate that policy support is needed to PrEP implementation and scale-up, such as decrease the cost of PrEP by supporting the development of generic PrEP drugs or cover some cost of PrEP by health insurance plans. Polices and projects targeting these barriers might facilitate optimizing the allocation of resources to promote PrEP in Chinese MSM.

## Supplementary Information

**Additional file 1: Table S1. **HIV incidence risk index for MSM (HIRI-MSM)^a^.

## Data Availability

The datasets are available from the corresponding authors on reasonable request.

## Abbreviations

MSM: Men who have sex with men
PrEP: Pre-exposure prophylaxis
ARVs: Antiretroviral drugs
WHO: World Health Organization
SPHCC: Shanghai Public Health Clinical Centre
STI: Sexually transmitted infection
nPEP: Nonoccupational post-exposure prophylaxis
HIRI: High-incidence risk index
TDF/FTC: Tenofovir/emtricitabine
ORs: Odd ratios
