# Supplementary material for: Multi-dimensional mismatch and barriers for promoting PrEP among men who have sex with men in China: a cross sectional survey from the Demand-side
Source: AIDS Res Ther. 2023 Feb 13;20:11. doi: 10.1186/s12981-022-00497-6 (PMC9926770; doi:10.1186/s12981-022-00497-6)
Supplement: Supplementary file 1 — Additional file 1: Table S1. HIV incidence risk index for MSM (HIRI-MSM)a. [file 12981_2022_497_MOESM1_ESM.docx]

**Supplementary Table 1. HIV Incidence Risk Index for MSM (HIRI-MSM)^a^**

| **Question number** | **Question** | **Response** | **Score** |
| --- | --- | --- | --- |
| 1 | How old are you today (years)? | <18 years | 0 |
|  |  | 18-28 years | 8 |
|  |  | 29-40 years | 5 |
|  |  | 41-48 years | 2 |
|  |  | >49 years | 0 |
| 2 | How many men have you had sex with in the last 6 months? | >10 male partners | 7 |
|  |  | 6-10 male partners | 4 |
|  |  | 0-5 male partners | 0 |
| 3 | How many of your male sex partners were HIV positive? | >1 positive partners | 8 |
|  |  | 1 positive partners | 4 |
|  |  | <1 positive partners | 0 |
| 4 | In the last 6 months, how many times did you have receptive anal sex (you were the bottom) with a man without a condom? | 1 or more times | 10 |
|  |  | 0 times | 0 |
| 5 | In the last 6 months, how many times did you have insertive anal sex (you were the top) with a man who was HIV positive? | 5 or more times | 6 |
|  |  | 0-4 times | 0 |
| 6 | In the last 6 months, have you used methamphetamines such as crystal or speed? | yes | 5 |
|  |  | no | 0 |
| 7 | In the last 6 months, have you used poppers (amylnitrate)? | yes | 3 |
|  |  | no | 0 |
